# Irreversible Electroporation for Tonsillar Ablation in Adults

**DOI:** 10.1002/ohn.70276

**Published:** 2026-04-30

**Authors:** Codrut Sarafoleanu, Ionut Tanase, Mihai Alexandru Pascu, Desiderio Passali, Ari DeRowe

**Affiliations:** ^1^ Department of ORL&HNS Sfanta Maria Hospital University of Medicine Carol Davila Bucharest Romania; ^2^ Department of ORL&HNS University of Medicine Carol Davila Bucharest Romania; ^3^ Department of ORL University of Siena UNISI ORL Siena Italy; ^4^ Pediatric Otolaryngology Unit, Otolaryngology Head and Neck Surgery Gray Faculty of Medical and Health Sciences, Tel Aviv Sourasky Medical Center Tel‐Aviv University Tel Aviv Israel

**Keywords:** electroporation, tissue ablation, tonsillectomy

## Abstract

**Objective:**

To evaluate the safety and efficacy of irreversible electroporation for non‐thermal ablation of tonsils in adults.

**Study Design:**

Prospective interventional case series of 24 adult patients referred for tonsillectomy that were followed for 3 months.

**Setting:**

Academic medical center.

**Methods:**

The procedures were performed under general anesthesia or sedation. Irreversible electroporation was applied to the tonsils using the ENTire^TM^ system. Pain was assessed daily using a pain scale for the first postoperative week. Tonsil sizes were assessed at baseline and 1 and 3 months postoperatively. The Tonsil and Adenoid Health Status Instrument and the Snore‐Visual Analog Scale (VAS) questionnaires were administered at baseline and at 3 months.

**Results:**

In total, 23 adult patients (mean age: 36.5 ± 14.0; mean body mass index [BMI]: 25.5 ± 4.1) completed the study. No bleeding was observed intra‐ or postoperatively. Procedural time was 7.98 ± 2.62 minutes. A pain scale score below 2 was reported 4.5 ± 1.7 days after intervention. Reduction in tonsillar size was observed from 2.4 ± 0.9 pre‐intervention to 1.3 ± 1.0 at 3 months post‐intervention (*P* < .001). Tonsil and Adenoid Health Status scores decreased from 17.7 ± 13.0 to 0.96 ± 1.7 (*P* < .001). The Snore‐ VAS score decreased by 72%.

**Conclusion:**

This is the first clinical study to investigate the use of irreversible electroporation for tonsillar ablation. Irreversible electroporation shows promise as a minimally invasive procedure for reducing the size of tonsillar hypertrophy and alleviating symptoms, with negligible bleeding and significantly less pain. Multicenter studies are in progress to evaluate long‐term efficacy including in children.

Electroporation is the application of a pulsed electrical field to tissue, resulting in the deactivation of cellular ion channels. Depending on the pulsed energy applied, the resulting pores in the cell membrane can allow for the influx of therapeutic molecules. At higher energy levels, pore formation becomes irreversible, leading to cellular apoptosis and subsequent tissue ablation. The main advantage of this technique is the precise application of the electrical field, which avoids damage to surrounding tissue and thus reducing the risk of complications.^1^ In addition, the non‐thermal nature of the procedure has the potential to reduce postoperative pain. The electrical pulses are delivered in a microsecond duration, resulting in a shorter procedure time. Irreversible electroporation (IRE) is increasingly being used for ablation in conditions such as liver cancer, cardiac arrhythmias, and prostate cancer.^2^, ^3^, ^4^ It offers a non‐thermal alternative to traditional ablation methods, reducing the risk of collateral damage to surrounding tissue.

Tonsillectomy is one of the most common procedures in otolaryngology. In the pediatric population, an estimated 559,900 ambulatory and 7100 inpatient tonsillectomies were performed in 2019.^5^ Among adults, approximately 297,000 tonsillectomies are performed annually.^6^ Complications following surgery, such as bleeding, are uncommon; however, there are concerns about an increased incidence of bleeding following tonsillectomy in adults, with a reported rate of 6.3% in recent years.^7^ Many adults experience severe and prolonged pain following tonsillectomy.^8^ There has been an increase in alternative surgical techniques, primarily involving partial or intracapsular approaches using various technologies such as mono/bipolar electrocoagulation,^9^ coblation,^10^ and microdebrider.^11^ These approaches aim to achieve similar clinical outcomes with reduced pain and fewer complications. However, evidence reported in a meta‐analysis has yet to show clear advantages of these techniques.^12^ The use of non‐thermal IRE technology for tonsillar hypertrophy has the potential to reduce pain and complications following tonsil ablation. This is the first study to investigate the use of an IRE system for tonsillar ablation in patients. The primary outcomes of interest in this study were safety and postoperative pain. Given the small and heterogeneous nature of the patient cohort, it would be inappropriate to draw conclusions regarding robust clinical efficacy. Nevertheless, to assess potential improvements in quality of life and obstructive symptoms, validated questionnaires were administered to all patients, irrespective of indication, both preoperatively and for follow‐up, as secondary outcomes. In addition, changes in tonsillar size following IRE were evaluated using the standard Brodsky scale.

## Materials and Methods

This is a prospective, single‐arm, non‐blinded study. Ethical approval was obtained from the Institutional Review Board of Sfanta Maria Hospital, Bucharest, as well as from the Romanian Ministry of Health (CIV‐ID: RO‐23_11_044856). Informed consent was obtained from each patient prior to the procedure. The study was registered at: https://clinicaltrials.gov/study/NCT06187194 “Efficacy and Safety of Irreversible Electroporation for Chronic Symptomatic Tonsillar Hypertrophy Treatment.”

### Study Participants

Twenty‐four otherwise healthy adult patients who were referred for tonsillectomy due to symptomatic tonsillar hypertrophy were invited to participate in the study. Tonsillar hypertrophy was defined by a Brodsky grade scale of tonsils (BGST) of more than 2+.^13^ Symptoms included recurrent or chronic infections and/or obstructive symptoms consistent with accepted guidelines.^14^

### Study Protocol

Preoperative evaluation included physical examination to determine BGST and completion of the two questionnaires: the Snore‐VAS and the Tonsil and Adenoid Health Status Instrument (TAHSI) as secondary outcome measures. For the primary outcome measure, following the procedure, all participants completed a daily VAS pain scale (0 = no pain to 10 = unbearable pain) via telephone interview. The first follow‐up visit was conducted at 1 week and included a physical examination. Additional follow‐up visits were conducted at 1 and 3 months, including physical examination to assess tonsil size using the BGST estimate, as well as administration of both the Snore‐VAS and the TAHSI. BGST scoring and the administration of the questionnaires were performed by an otolaryngology resident in training that was not one of the principal investigators.

### The IRE Procedure

**Figure 1:**
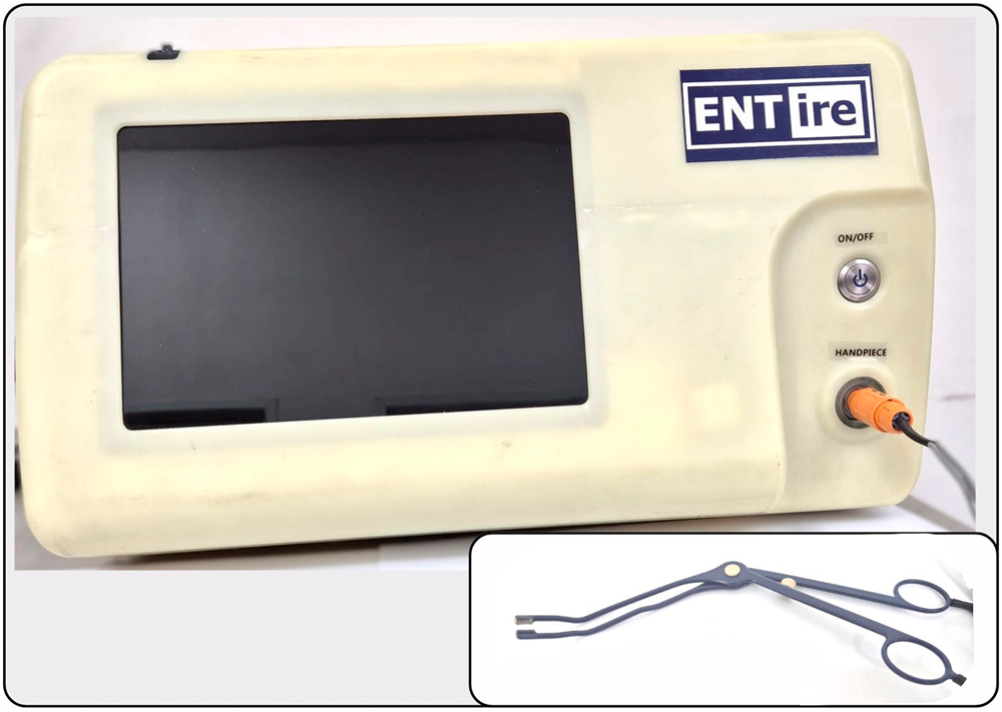
ENT‐ire irreversible electroporation (IRE) generator and tonsil handpiece.

The procedure was performed under general anesthesia or sedation with local anesthesia, depending on patient and surgeon preference. The IRE System (ENTire Inc., Delaware, USA) consists of a generator (console) and a passive handpiece designed to deliver high‐voltage electric power to the tissue by generating suitable electric fields to induce the IRE effect (Figure 1). The generator features a user‐friendly interface that allows the user to monitor system status and control parameters. A foot pedal connected to the generator is used to operate the system. The user interface is linked to a control unit that controls the high‐voltage power supply and activates the system. The pulse generator is connected to an output module. The handpiece is shaped like bipolar forceps with two distal medical‐grade stainless steel electrodes that deliver electrical energy to the tissue. The electrodes grab the tissue without penetration. Following the procedure, for pain management, an non‐steroidal anti‐inflammatory drugs was prescribed PRN.

### Pain Assessment and Questionnaires

The pain scale ranged from 0, indicating no pain, to 10, indicating excruciating pain.^15^ The Snore‐VAS consisted of three questions: Q1: “How frequently do you snore?” Q2: “How much does your snoring bother you?” Q3: “If relevant, how much does your snoring bother your partner?”^16^ The TAHSI is a validated quality of life questionnaire related to tonsil and adenoid disease. Although primarily used in children, it has also been validated in adults.^17^ The questionnaire consists of 15 items grouped into 6 subscales: eating and swallowing, airway and breathing, infections, healthcare utilization, cost of care, and behavior. Each item is rated on a 5‐point Likert scale.

### Statistical Analysis

All measured variables and derived parameters were tabulated by descriptive statistics. For categorical variables, summary tables are provided giving sample size, absolute and relative frequency, and 95% CI (Clopper Pearson Confidence Interval) for proportions. For continuous variables, summary tables are provided giving sample size, arithmetic mean, standard deviation, minimum, median, and maximum. The signed‐rank test was applied to test the significance of the changes/values relative to the criteria of success for each parameter (when applicable). All tests are two‐tailed, and a *P* value of 5% or less was considered statistically significant. Data were analyzed using SAS version 9.4 (SAS Institute).

## Results

Twenty‐four patients were included in the study. One patient did not return for the 1‐month follow‐up and was therefore excluded from the analysis. The final sample comprised 23 patients otherwise healthy (mean age: 36.5 ± 14.0; mean body mass index [BMI]: 25.5 ± 4.1). Eleven male, 13 female. The procedure was performed under general anesthesia in 14 patients and under sedation with local anesthesia in 9 patients. There were no differences in baseline or outcome measures regarding anesthesia modality.

No complications occurred during any of the procedures. The net procedural time was 7.98 ± 2.62 minutes. No intraoperative bleeding was reported.

**Figure 2:**
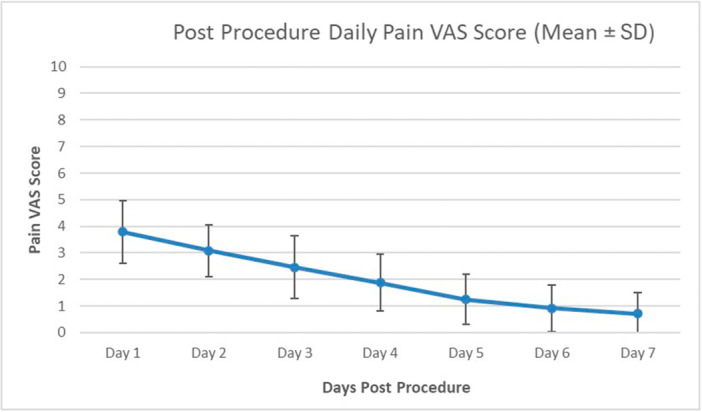
Daily visual analog scale (VAS) pain scores postoperative days 1 to 7.

The mean postoperative pain at 1‐week post‐surgery was 1.15 ± 0.88. Figure 2 shows the decrease in pain scores during the first week after surgery.

Table 1 shows the reduction in BGST size for 46 tonsils at 3 months following surgery in comparison to their size at baseline. Three months after surgery, BGST size was reduced by an average of 52.54% ± 22.83%. Of note, 96% of tonsils showed one or greater than 1 BGST grade reduction at 3 months after surgery (95% confidence interval of 78.9%, 99. 9%, Wilcoxon signed‐rank test *P*‐value of <.0001).

**Table 1:** Change in Brodsky Grade Scale of Tonsils (BGST) at 3 Months Post‐treatment Compared to Baseline

BGST mean ± SD	Baseline	3 mo	Δ 3 mo to baseline	Δ% 3 mo to baseline
N of tonsils	48	46	46	46
Left	2.54 ± 0.78	1.35 ± 0.83	−1.13 ± 0.34	−48.91% ± 19.35%
Right	2.50 ± 0.78	1.17 ± 0.94	−1.26 ± 0.45	−56.16% ± 25.77%
Total	2.52 ± 0.77	1.26 ± 0.88	−1.20 ± 0.40	−52.54% ± 22.83%

Table 2 demonstrates the change in the Snore‐VAS and TAHSI scores at 3 months compared to baseline. Significant reductions in both scores were observed 3 months after surgery. The Snore‐ VAS score decreased by 72% and the TAHSI by 93% at 3 months following surgery in comparison to baseline. Table 3 shows the change in scoring of the TAHSI sub‐categories from baseline to follow‐up. The highest scores at baseline were for infection and healthcare utilization, which were reduced significantly in 3 months. Regarding the remaining sub‐categories, the baseline scoring was low, so the reduction in scoring is not clinically relevant.

**Table 2:** Change in Snore‐Visual Analog Scale (VAS) and Tonsil and Adenoid Health Status Instrument (TAHSI) Scores at 3 Months Post‐surgery in Comparison to Baseline

	Baseline	3 mo	Δ 3 mo to baseline	Δ% 3 mo to baseline
N	24	23	23	23
Snore‐VAS (mean ± SD)	10.43 ± 8.27	2.68 ± 3.34	−7.45 ± 7.09	−72.07% ± 2.03
TAHSI (mean ± SD)	17.71 ± 3.02	1.00 ± 1.71	−15.26 ± 11.31	−93.22% ± 2.23%

**Table 3:** Changes in Tonsil and Adenoid Health Status Instrument (TAHSI) Scores for Each Component

TAHSI subscale (Mean ± SD)	Baseline	3 mo	Δ 3 mo to baseline	Δ% 3 mo to baseline
N	23	23	23	23
Airway and breathing	2.2 ± 3.3	0.7 ± 1.4	−1.6 ± 2.6	−70.6%
Infection	5.0 ± 3.1	0.1 ± 0.3	−4.9 ± 3.1	−98.2%
Healthcare utilization	5.6 ± 3.5	0.2 ± 0.6	−5.4 ± 3.5	−96.9%
Eating and swallowing	2.7 ± 4.8	0.0 ± 0.2	−2.6 ± 4.8	−98.4%
Cost of care	0.3 ± 0.6	0.0 ± 0.0	−0.3 ± 0.6	−100%
Breath	0.5 ± 1.1	0.0 ± 0.2	−0.5 ± 1.1	−91.7%

## Discussion

This is the first study to report the safety and efficacy of a new IRE‐based technology for tonsillar ablation. We show the potential advantage of non‐thermal IRE ablation through a reduction in post‐procedural pain, likely due to preservation of surrounding tissue and minimal mucosal damage. The postoperative course was uncomplicated, with relatively low pain scores on day 1 and minimal pain by day 7 post‐surgery. There were no complications.

Tonsillectomy is a common surgical procedure, effective in treating airway obstruction caused by tonsillar hypertrophy and in managing recurrent/chronic infections. The primary outcomes of interest in this study were safety and postoperative pain. Nevertheless, to assess potential improvements in quality of life and obstructive symptoms, validated questionnaires were administered to all patients, irrespective of indication, both preoperatively and for follow‐up. In addition, changes in tonsillar size following IRE were evaluated using the standard Brodsky scale. Following tonsillectomy, patients often experienced prolonged and sometimes severe postoperative pain. The results of the present study show the potential of the non‐thermal IRE ablation technique in reducing pain. In comparison with the literature, postoperative pain following tonsillectomy is typically reported to range from 5.6 to 5.8 on a 0 to 10 numerical rating scale.^18^ In contrast, the mean postoperative pain in our study was 4 on postoperative day 1. By postoperative day 7, reported pain scores following tonsillectomy generally range from 3 to 3.5, whereas mean pain scores in our cohort were below 1.

In adults, the rate of post‐tonsillectomy bleeding is approximately 6%,^6^, ^7^ and in many cases, it requires returning to the operating room. In rare instances, bleeding can be fatal. IRE holds promise in this regard, as tissue is not physically disrupted. In this study, although only a small number of patients were treated, no bleeding was observed intraoperatively or postoperatively.

In recent years, there has been a shift away from conventional tonsillectomy techniques toward less invasive options, such as coblation and intracapsular approaches.^19^ Although these procedures have reduced pain and bleeding complications, this reduction has not been substantial. A meta‐analysis comparing intracapsular and extracapsular tonsillectomy, which included 12 studies, found that the return to a normal diet improved by one day for intracapsular tonsillectomy, with the recovery time reducing from 5 days for extracapsular tonsillectomy to 4 days for intracapsular. This difference was statistically significant. However, no significant difference was observed between the two techniques in terms of postoperative bleeding, either primary or secondary.^11^

IRE is an emerging technique for ablating cardiac arrhythmias and solid tumors.^20^ Its key advantage over thermal methods is the absence of heat, preserving nearby nerves, vessels, and mucosa, thereby reducing invasiveness and postoperative pain. Histologically, IRE induces delayed apoptosis while maintaining tissue architecture, which likely contributes to lower pain levels. The results of the current study showed a low level of pain postoperatively and a significant reduction in tonsillar size and improvement in symptoms. The main limitation of the present study is the small sample size and heterogeneous patient population. Therefore, conclusions regarding clinical efficacy could not be established based on this data. However, the findings of improvement in subjective measures of health in this study are promising. In addition, the energy parameters such as pulse duration, number of consecutive pulses, number of applications, and voltage are still to be determined for optimal results. This study followed patients for 3 months, which was adequate to demonstrate safety and effectiveness; however, long‐term follow‐up is necessary to address issues of tonsillar regrowth and recurring symptoms.

Notwithstanding these considerations, the findings of the present study are promising, particularly with respect to reduced postoperative pain and the absence of bleeding. These advantages of non‐thermal IRE ablation are especially relevant in the pediatric population, where improving safety and reducing postoperative morbidity associated with adenotonsillectomy are especially important. Clinical studies in children are currently underway to address this population. Larger, multicenter studies are required to confirm and validate these preliminary findings in both adults and children.

## Author Contributions

**Codrut Sarafoleanu,** conceptualization, investigation, methology, project administration, supervision, validation, writing review and editing; **Ionut Tanase,** data curation, investigation, writing—review and editing; **Michai Alexandru,** data curation, investigation, writing—review and editing; **Desiderio Passali,** conceptualization, writing—review and editing; **Ari DeRowe,** conceptualization, methology, formal analysis, supervision, validation, writing—original draft.

## Disclosures

### Competing interests

Ari DeRowe is on the medical advisory board of ENTire Inc.

### Funding source

The study was funded by ENTire Inc.
